# A New Mechanism of Silicone Oil-Induced Glaucoma and Its Management

**DOI:** 10.1155/2022/2343139

**Published:** 2022-06-02

**Authors:** Harsh Kumar, Dinesh Talwar, Mithun Thulasidas, Surbi Taneja

**Affiliations:** ^1^Glaucoma Services, Centre for Sight, B-5/24, Safdarjung Enclave, New Delhi, India; ^2^Vitreoretinal Services, Centre for Sight, B-5/24, Safdarjung Enclave, New Delhi, India

## Abstract

**Purpose:**

To describe a case of secondary acute angle closure glaucoma due to silicone oil migration into the posterior chamber causing entrapment of aqueous and its successful management. *Case Presentation*. A 69-year-old female presented with decreased vision and pain in the left eye (LE) for one month. She had a history of complicated phacoemulsification with nucleus drop and retinal detachment in LE, for which vitreoretinal surgery with silicone oil endotamponade was done. She was also a known case of primary open angle glaucoma on medications. The corrected distance visual acuity was 20/20 and 20/125 in the right eye (RE) and LE, respectively. The intraocular pressure (IOP) was 18 mmHg in RE and 45 mmHg in LE. Anterior segment examination of LE revealed 270° of iridocorneal apposition in the periphery of the anterior chamber. Fundus examination of LE showed silicone oil filled vitreous cavity with attached retina. Given the recent history of silicone oil injection and elevated IOP despite maximum antiglaucoma medications, we decided to perform laser peripheral iridotomy (LPI) in the area of iridocorneal apposition. Following LPI, the IOP in LE came down to 17 mmHg and remained stable within the normal range for one month, after which the patient was taken up for silicone oil removal.

**Conclusion:**

This case report highlights a new mechanism of silicone oil-induced glaucoma and the technique of performing LPI in the area of iridocorneal apposition, for the first time in the literature. Silicone oil migration into the posterior chamber from the vitreous cavity in the presence of zonular dehiscence can push the iris forward and lead to iridocorneal apposition, resulting in an acute rise in IOP. Performing LPI within the area of iridocorneal apposition can help the aqueous seep into the anterior chamber and release the silicone oil globule trapped behind the iris to enter the anterior chamber, thus relieving the iridocorneal adhesions and lowering the IOP.

## 1. Introduction

Secondary glaucoma after vitreo-retinal (VR) surgery with silicone oil injection is a relatively common complication. Secondary glaucoma can develop at any time in the postoperative phase and can manifest with a wide range of intraocular pressure (IOP) and loss of vision. Silicone oil is a vitreous substitute used for long-term intraocular tamponade in VR surgery, usually for a period of 3-6 months, depending on the viscosity of silicone oil, retinal detachment, and surgeon's choice. Silicone oil may be left for a long-term period also in a few cases. Two main types of silicone oil available are low viscosity oil (lighter than water) and high viscosity oil (heavier than water). Low viscosity oil floats in the eye, and high viscosity oil provides long-term tamponade for inferior retinal pathologies as the oil sinks in the vitreous cavity [1–3].

Secondary glaucoma following silicone oil injection can occur due to different mechanisms, including pupillary block, migration of silicone oil into the anterior chamber, inflammation, preexisting glaucoma, trabecular meshwork infiltration by silicone oil bubbles, synechial angle closure, and rubeosis iridis [4–8].

Here, we describe a case of silicone oil-induced glaucoma due to a new mechanism and its successful management.

## 2. Case Presentation

A 69-year-old female presented to our outpatient services with decreased vision, pain, and discomfort in the left eye (LE) for one month. She had a history of phacoemulsification with intraoperative posterior capsule rupture and nucleus drop into the vitreous cavity in the LE, for which pars plana vitrectomy followed by nucleus removal (using a fragmatome) was performed, and a three-piece intraocular lens (IOL) was implanted in the ciliary sulcus a month ago. During the postoperative period, the patient developed retinal detachment, for which she underwent VR surgery with silicone oil endotamponade. The patient was also a known case of open angle glaucoma on four antiglaucoma medications (Latanoprost 0.005%, Timolol Maleate 0.5%, Brimonidine Tartrate 0.2%, and Ripasudil 0.4%) in both eyes prior to the cataract surgery. She was asked to stop Latanoprost 0.005% eye drops in LE one week before the surgery and continued the rest of the medications.

**Figure 1:**
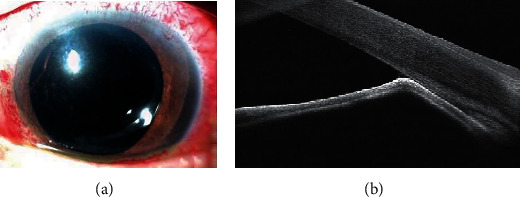
(a) Anterior segment photograph of the left eye showing 270° of iridocorneal apposition in the periphery of the anterior chamber. (b) Anterior segment optical coherence tomography of the left eye showing iridocorneal apposition.

On examination, the corrected distance visual acuity (CDVA) was 20/20 and 20/125 in the right eye (RE) and LE, respectively. The IOP was 18 mmHg in RE and 45 mmHg in LE on Goldman applanation tonometry (GAT). Anterior segment examination of RE revealed in-the-bag IOL. LE showed circumcorneal congestion, mild corneal epithelial edema, 270° of iridocorneal apposition in the periphery of the anterior chamber 2-3 mm from the limbus, and in-the-sulcus IOL (Figure 1(a)). No silicone oil in the anterior chamber was observed. Fundus examination showed a cup-disc ratio (CDR) of 0.8 with bipolar neuroretinal rim thinning, normal macula, and attached retina in RE and a CDR of 0.6 with inferior neuroretinal rim thinning with silicone oil-filled vitreous cavity and attached retina in LE. Preoperative visual fields with a Humphrey Field Analyzer 3 24-2 Swedish Interactive Thresholding Algorithm Standard programme showed a visual field index of 51% in RE and 87% in LE. Central corneal thickness was 536 *μ* and 505 *μ* in RE and LE, respectively. Iridocorneal apposition was observed on anterior segment optical coherence tomography (AS-OCT) of LE (Figure 1(b)). A diagnosis of secondary angle closure due to silicone oil in LE was made.

The patient was given 200 cc intravenous mannitol 20% and was started on maximum antiglaucoma medications adding Pilocarpine 2% eye drops thrice daily, Latanoprost 0.05% at bedtime in LE, and oral acetazolamide 250 mg three times a day with potassium supplementation, along with the previous medications. The next day, she had an IOP of 40 mmHg in LE on GAT. Syrup Glycerol (50%, 30 ml) thrice a day was also added. She still had an IOP of 36 mmHg in LE on the following day. The choice to remove the silicone oil either partially or completely was discussed with the VR surgeon. However, as the surgery had been done only a few days back, this posed the risk of redetachment of the retina. Hence, silicone oil removal was deferred.

**Figure 2:**
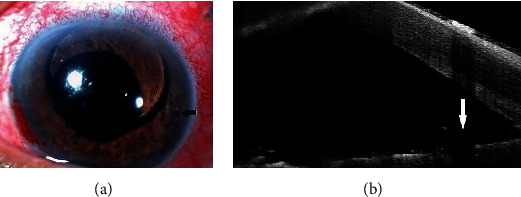
(a) Anterior segment photograph of the left eye showing seeping of silicone oil into the anterior chamber following laser peripheral iridotomy (black arrow). (b) Anterior segment optical coherence tomography of the left eye showing the release of iridocorneal apposition and widening of the angle following laser peripheral iridotomy (white arrow).

As the entrapment of silicone oil between the IOL and the iris was the cause for iridocorneal apposition, we considered the possibility of performing a laser peripheral iridotomy (LPI) in the area of iridocorneal apposition, which might permit egress of aqueous into the anterior chamber. LPI was performed in the area of iridocorneal apposition on the temporal aspect between 3 and 4 o'clock, which led to the seeping of aqueous through the PI into the anterior chamber, relieving the iridocorneal apposition over the entire angle. The deepening of the anterior chamber and posterior shift of the iris was followed by the entry of the silicone oil into the anterior chamber through the pupil, where it formed a globule of around 6 mm diameter in the anterior chamber (Figure 2(a)). One hour later, the IOP in LE came down to 17 mmHg, and AS-OCT showed the release of iridocorneal apposition (Figure 2(b)). The IOP remained stable within the normal range on consecutive follow-ups for one month, after which the patient was taken up for silicone oil removal.

### 2.1. LPI Technique for Iridocorneal Apposition

Pilocarpine 2% eye drops were used to cause miosis, stretch the iris, and facilitate perforation. After instilling Proparacaine 0.5% eye drops, Abraham iridotomy lens was applied to the eye with a coupling gel. Neodymium-doped yttrium aluminium garnet (Nd:YAG) laser was used to create LPI. The aiming beam was defocused to 125 *μ* posterior focus and was then focused within the iris stroma in the area of iridocorneal apposition. The part of iris tissue between its original plane and its point of apposition with the cornea was focused at the slight recess seen in the AS-OCT in the extreme periphery.

The initial shot was made in the slight recess at the extreme peripheral area with 2 mJ of energy which helped push back the iris further away from the cornea due to the force of plasma formation. Immediately, the energy was increased to 4 mJ, and partial patency of the iridotomy was achieved. After the slight deepening due to some egress of aqueous, 6 mJ energy was used to complete the iridotomy.

## 3. Discussion

The incidence of elevated IOP or glaucoma following silicone oil injection varies from 2.2% to 56.0% [3, 9]. Multiple mechanisms of silicone oil-induced secondary glaucoma have been described in the literature [4–8]. In the present case, we have described a new mechanism of silicone oil-induced angle closure glaucoma and how we managed it.

Usually, overfill of silicone oil in the vitreous cavity, when associated with partial zonular dehiscence, results in the anterior movement of silicone oil into the anterior chamber. If the anterior chamber is completely filled with silicone oil, direct closure of the angle and resultant glaucoma can occur, even with intact zonules. Removal of silicone oil from the anterior chamber and partial removal from the vitreous cavity is the management option in such situations.

However, in our case, we believe that the silicone oil globule was trapped in the posterior chamber and pushing the iris forward, leading to iridocorneal touch in the periphery and a physical block of the anterior chamber angle. The silicone oil bubble trapped in the posterior chamber also possibly caused an occult pupillary block due to its surface tension preventing egress of aqueous into the anterior chamber. This possibly may have exacerbated the peripheral iridocorneal touch even further. The angle was closed 2-3 mm from the limbus temporally and caused flattening of the anterior chamber in the temporal quadrant. As the damming of the aqueous in the posterior chamber increased, the iridocorneal touch also developed in the nasal quadrant. This caused a hindrance to aqueous outflow as the conventional outflow system got blocked at the level of entry of aqueous into the trabecular meshwork. Consequently, this led to an increase in IOP. No apparent pupillary block and no iris bombe were present.

It seems likely that the silicone oil, which was injected at the end of VR surgery, partially seeped into the posterior chamber through an area of zonular dehiscence that occurred during the previous cataract surgery or VR surgery [2]. The high viscosity of silicone oil resulted in the entrapment of silicone oil behind the iris and is likely to have been aggravated by the collection of aqueous in the posterior chamber, which pushed the peripheral iris forward and flattened the anterior chamber in the periphery. This flattening further prevented the escape of silicone oil into the anterior chamber.

A prophylactic inferior surgical iridectomy is absolutely indicated in aphakic patients with silicone oil endotamponade due to the high risk of pupillary block [10]. In phakic and pseudophakic patients, there is no role of prophylactic iridectomy or iridotomy usually as the risk of silicone oil pupil block is rare [5]. However, in cases of blunt trauma or cataract surgery with subclinical zonular loss and extensive vitreous base excision, displacement of silicone oil and pupillary block could occur [11–13]. In the present case, surgical iridectomy was not done as there was no reason to believe that there was a significant loss of zonular integrity, and no air entered the anterior chamber during the fluid-air exchange, which could have suggested the likelihood of the possibility of zonular dialysis. Anterior movement of silicone oil is most likely to occur in a patient with zonular dehiscence if the patient fails to sleep in the prone position. Hence, a prophylactic surgical iridectomy or iridotomy shall be considered in cases where zonular loss is likely.

LPI is the commonly performed procedure to relieve an acute angle closure attack and to relieve the raised IOP in eyes with iris bombe due to secondary angle closure [14]. Patients with chronic unrelieved angle closure can often develop peripheral anterior synechiae, which cause closure of the trabecular meshwork and prevent aqueous outflow, thereby resulting in elevated IOP. Similarly, cases with extensive iridocorneal contact in the periphery can lead to raised IOP due to blockage of the angle. Performing LPI in an almost apposed iridocorneal situation is challenging and usually not done. The technical hitch in performing LPI in the presence of iridocorneal touch is the likelihood of localised corneal damage and the inability to complete the procedure in a nonexistent space.

However, in our case, we have decided to perform LPI in the area of iridocorneal apposition, as the patient was already on maximum antiglaucoma medications and creating a PI was the only solution to relieve the symptoms of the patient and prevent further damage to the optic nerve head. A low energy of 2 mJ was used in the initial shot, which helped push back the iris away from the cornea, resulting in our ability to increase the energy to create full penetration of the iris. After the creation of a PI in the region of iridocorneal adhesion, it was noted that the aqueous had begun to seep into the anterior chamber through it. This deepened the anterior chamber and squeezed the silicone oil trapped between the iris and the IOL, through the pupil into the anterior chamber. The high viscosity of the silicone oil, which initially prevented its movement through the pupil, now facilitated its movement when the posterior chamber of the eye between the IOL and the iris flattened. This also relieved the iridocorneal adhesions at the level of the midperiphery of the cornea. The deepening of the anterior chamber allowed further enlargement of the size of PI. The creation of an alternative passage for the flow of aqueous in the presence of an occult obstruction caused by silicone oil leads to a decrease in IOP. This outcome suggests that the passage for aqueous flow through the trabecular meshwork opened up as the iridocorneal apposition was fresh, and only the route of aqueous entry into the angle was blocked.

To the best of our knowledge, this is the first case report to describe a new mechanism of silicone oil-induced angle closure glaucoma and the unique technique of performing LPI in the area of iridocorneal apposition. Our case highlights that the clinicians should be aware of the mechanism described, and a simple procedure like LPI can give time to let the retinal reattachment become stable enough for the silicone oil to be removed from the vitreous cavity.

## Data Availability

The data that support the findings of this case report are available in the report.
